# Nanoparticles for Drug Delivery: Design, Mechanisms, and Clinical Translation

**DOI:** 10.3390/molecules31173121

**Published:** 2026-09-06

**Authors:** Subin Antony Jose, Benjamin Crutchfield, Mario Caballero, Eadrian Carreon, Ian Davis, Pradeep L. Menezes

**Affiliations:** Department of Mechanical Engineering, University of Nevada, Reno, NV 89557, USA; subinj@unr.edu (S.A.J.); eadrianc@unr.edu (E.C.); iandavis@unr.edu (I.D.)

**Keywords:** nanoparticles, drug delivery, nanomedicine, polymeric nanoparticles, clinical translation, artificial intelligence, green nanomanufacturing

## Abstract

Nanoparticle-based drug delivery systems have become an important component of modern nanomedicine, enabling improved drug protection, controlled release, targeted delivery, and the modulation of pharmacokinetic behavior. Their therapeutic performance is governed by physicochemical properties such as size, shape, surface chemistry, and material composition, which influence biological interactions, biodistribution, cellular uptake, and clearance. This review examines major nanoparticle platforms, including polymeric, lipid-based, inorganic, carbon-based, and hybrid systems, together with passive and active targeting and endogenous and externally triggered release strategies. Current and emerging applications in oncology, infectious diseases, central nervous system disorders, gene therapy, and vaccines are discussed alongside theranostic and combination-delivery approaches. Particular emphasis is placed on computational modeling, artificial intelligence, and digital twins for formulation optimization and personalized nanomedicine. Key barriers to clinical translation, including manufacturing scalability, biological variability, limitations of EPR-mediated targeting, regulatory standardization, and long-term safety, are critically evaluated. Finally, emerging directions in sustainable nanomanufacturing and biomimetic delivery are discussed. By integrating biological mechanisms with computational, manufacturing, regulatory, and clinical considerations, this review provides a translational perspective on advancing nanoparticle drug-delivery systems from laboratory development toward clinical implementation.

## 1. Introduction

### 1.1. From Conventional Systems to Nanomedicine: Needs and Opportunities

The development of nanomedicine has been driven by the accumulating limitations of conventional drug delivery approaches, such as oral tablets, capsules, and parenteral injections, which were adequate for small-molecule pharmaceuticals but are proving increasingly insufficient for the complex, fragile, and highly potent therapeutic agents that dominate modern pipelines. Standard dosage forms suffer from poor aqueous solubility, rapid systemic clearance, nonspecific biodistribution, and resulting off-target toxicity, which reduces therapeutic index and treatment tolerability [1]. In oncology, cardiovascular diseases, and neurological disorders, these limitations are particularly consequential. Drugs must reach a localized disease site at therapeutic concentrations while sparing surrounding healthy tissue, a spatial discrimination that conventional formulations cannot achieve. The progressive increase in the molecular complexity of biologics, nucleic acid therapeutics, and precision oncology agents has further widened the gap between drug potency and delivery capability, as many of these agents are inherently unstable in biological fluids, membrane-impermeable, or rapidly degraded before reaching the target cells [2,3].

Compared with conventional pharmaceutical formulations, nanoparticle-based delivery systems can provide additional control over drug solubility, protection from premature degradation, pharmacokinetics, biodistribution, and site-specific or stimulus-responsive release. These advantages are particularly relevant for poorly soluble drugs and biologics such as proteins and nucleic acids; however, they are accompanied by greater formulation complexity, manufacturing and characterization requirements, potential nanomaterial-specific safety concerns, and additional regulatory challenges [4,5].

Nanomedicine broadly refers to the application of nanoscale materials and technologies to the diagnosis, prevention, monitoring, and treatment of disease, including their use as engineered systems for therapeutic delivery. Within this field, nanoparticles are generally defined as materials or structures with dimensions in the nanoscale range, although pharmaceutical nanocarriers may extend beyond the conventional 1–100 nm definition depending on their composition and intended biomedical function [6]. In drug delivery, these systems are engineered to encapsulate, protect, transport, and release therapeutic agents while modifying their pharmacokinetic and biodistribution profiles [7]. Nanoparticles below approximately 200 nm exhibit preferential accumulation in tumor vasculature through the EPR effect, the passive extravasation of nanocarriers through fenestrated tumor blood vessels, and their retention due to impaired lymphatic drainage [3]. Beyond this passive mechanism, nanoparticles can be equipped with active targeting ligands, antibodies, peptides, and aptamers that selectively bind disease-associated receptors, enabling discrimination between diseased and healthy tissue at the molecular level [2]. The resulting combination of extended circulation, site-specific accumulation, and controlled release within the therapeutic target constitutes the central value proposition of nanomedicine over conventional dosage forms.

### 1.2. Core Advantages and Inherent Challenges

The advantages of nanoparticle-based drug delivery extend across multiple dimensions of therapeutic performance. Protection of labile cargo (nucleic acids, proteins, and unstable small molecules) from enzymatic degradation during systemic circulation is one of the most clinically significant benefits, enabling the delivery of therapeutic classes that were previously considered undruggable due to stability limitations [8]. Controlled and sustained drug release over hours to weeks reduces peak plasma concentration fluctuations, maintains drug levels within the therapeutic window for prolonged periods, and decreases dosing frequency, all of which improve both therapeutic consistency and patient adherence [9,10]. The multifunctionality of modern nanoparticle platforms, their ability to co-load multiple therapeutic agents at defined molar ratios, incorporate imaging reporters, and carry targeting moieties simultaneously, enables combination therapies, theranostics, and sequential drug release strategies that are structurally impossible with conventional single-molecule formulations [11,12].

Despite these advantages, several fundamental challenges continue to limit the broader clinical translation of nanomedicine. The complexity of nanoparticle systems introduces variability in synthesis, characterization, and batch reproducibility that is difficult to control at the manufacturing scale [13]. Protein corona formation (the rapid adsorption of serum proteins onto nanoparticle surfaces upon systemic injection) alters targeting ligand accessibility, accelerates immunological recognition, and modifies pharmacokinetic behavior in ways that are poorly predicted by in vitro testing [14,15]. Long-term biodistribution and organ accumulation of non-biodegradable materials, particularly inorganic nanoparticles, remain incompletely characterized, raising safety questions that regulatory agencies require extensive preclinical evidence to address [16,17]. Regulatory frameworks continue to evolve in response to the technological diversity of nanomedicines, and the absence of globally harmonized standards for nanoparticle characterization and safety assessment creates friction in the approval process [18]. Overcoming these barriers requires not only advances in materials science and formulation engineering, but also progress in manufacturing technology, computational design, and regulatory science.

Unlike conventional reviews that primarily classify nanoparticle platforms or summarize their biological applications, this review integrates therapeutic design with an engineering and translational perspective. Particular emphasis is placed on the convergence of AI-guided formulation design and digital twins, scalable and sustainable nanomanufacturing, and the regulatory and production challenges governing clinical translation. By connecting nanoparticle design, biological performance, computational optimization, manufacturing, and clinical implementation within a unified framework, this review aims to bridge the gap between laboratory-scale innovation and clinically deployable nanomedicines.

### 1.3. Scope and Organization of the Review

This review critically examines nanoparticle-based drug delivery from physicochemical design and biological interactions to therapeutic applications and clinical translation. Unlike reviews focused primarily on nanoparticle classification or biological applications, this review emphasizes the integration of AI-guided formulation design and digital twins, scalable and sustainable nanomanufacturing, and the engineering, safety, and regulatory considerations governing clinical translation. By connecting nanoparticle design, biological performance, computational optimization, manufacturing, and clinical implementation, the review aims to bridge the gap between laboratory-scale innovation and clinically deployable nanomedicines.

## 2. Fundamentals of Nanoparticle Design and Biological Interactions

### 2.1. Physicochemical Properties: Size, Shape, Surface Chemistry, and Drug Loading

**Figure 1:**
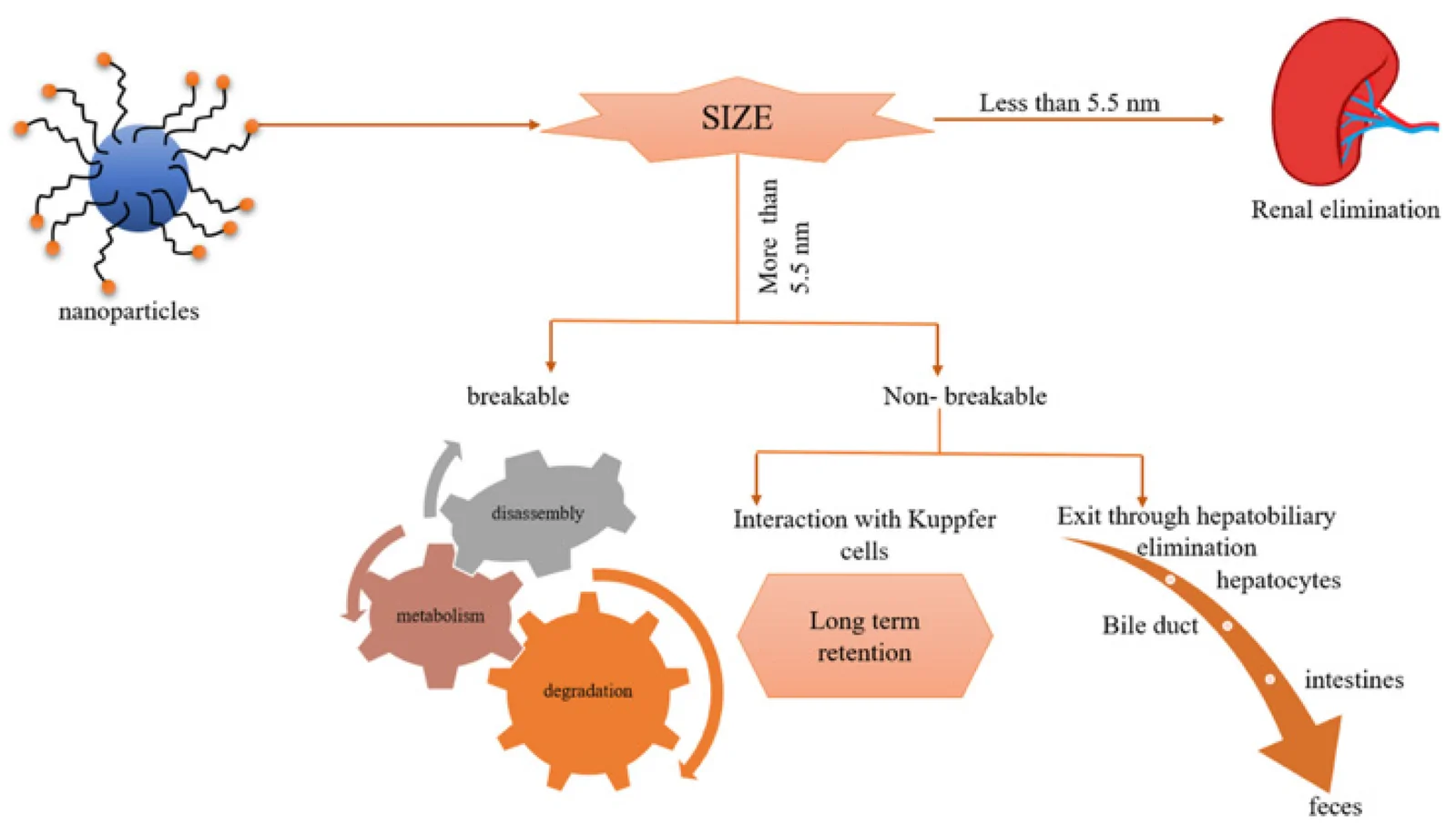
Effect of nanoparticle size on circulation behavior, biodistribution, tissue penetration, and clearance mechanisms in vivo, illustrating the size-dependent transition between renal elimination, prolonged systemic circulation, and uptake by the mononuclear phagocyte system. Reprinted from Ref. [21], under the terms of the Creative Commons CC BY license.

The biological behavior of drug-delivery nanoparticles is governed by the interplay between their material composition, structural architecture, and physicochemical properties. The systems considered in this review include polymeric nanoparticles and micelles, lipid-based nanoparticles and liposomes, inorganic nanoparticles such as silica, gold, and iron oxide systems, carbon-based nanomaterials, and hybrid or biomimetic carriers. These platforms differ structurally, ranging from solid or porous matrices and inorganic cores to micellar assemblies and lipid bilayer vesicles, and these differences influence drug loading and release, protein adsorption, cellular interactions, biodistribution, and clearance [19]. Among their physicochemical characteristics, particle size is a particularly important determinant of circulation, tissue distribution, cellular uptake, and elimination. In general, larger nanoparticles (>200 nm) are more susceptible to recognition and clearance by the mononuclear phagocyte system (MPS), whereas nanoparticles with hydrodynamic diameters below approximately 5.5 nm, particularly small inorganic systems, may undergo efficient renal filtration and urinary excretion [7]. The optimal size window for passive tumor targeting via the EPR effect is generally cited as 10–200 nm, though the actual effective range varies with tumor type, vascularization density, and disease stage. Within this range, smaller particles penetrate deeper into tumor tissue, while larger particles may accumulate at the periphery [20]. Figure 1 summarizes the influence of nanoparticle size on biological fate and pharmacokinetic behavior in vivo.

**Figure 2:**
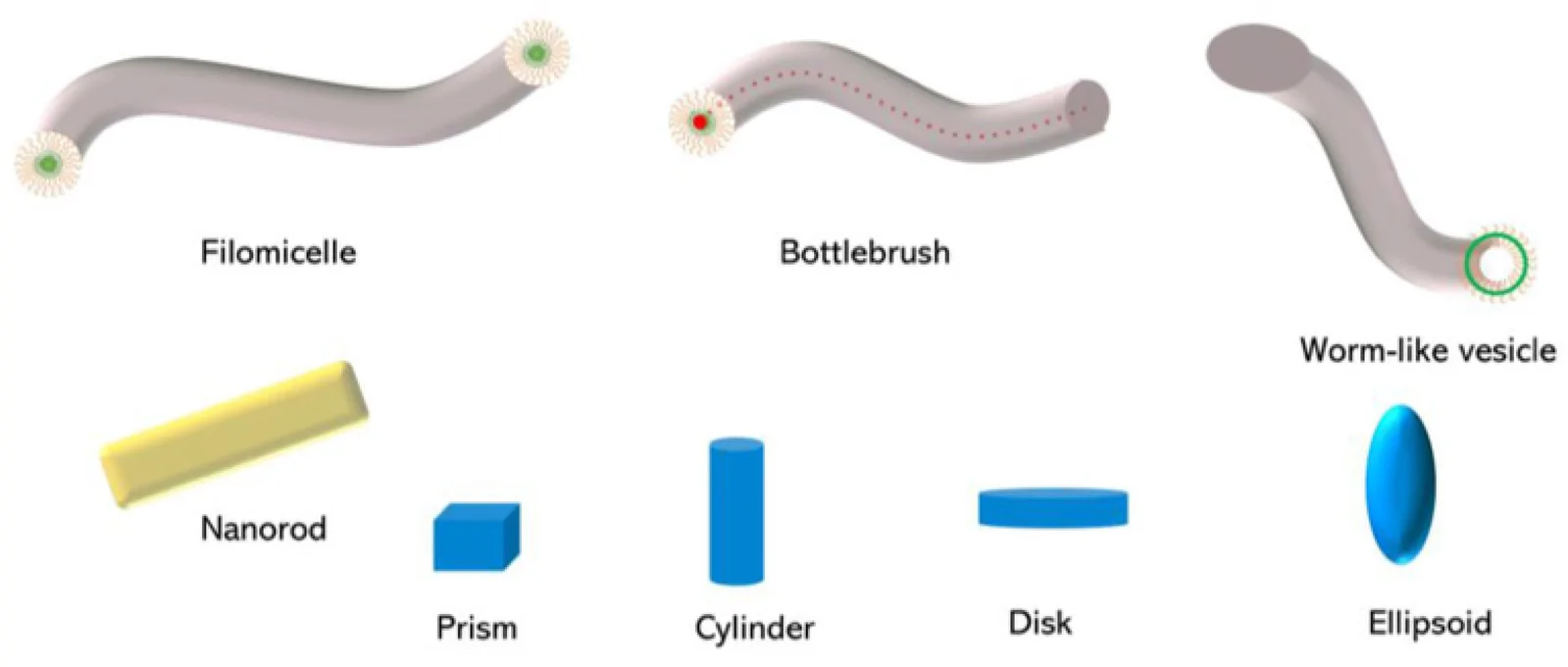
Representative non-spherical nanoparticle geometries used in drug delivery applications. Reprinted from Ref. [24], under the terms of the Creative Commons CC BY license.

Shape is an equally important but often underappreciated design variable. Spherical nanoparticles are the most widely studied geometry and are favored for their predictable behavior and efficient endocytosis, but non-spherical morphologies confer distinct biological advantages. Rod-shaped particles exhibit prolonged circulation times relative to spheres of equivalent volume, attributed to their reduced phagocytic uptake due to asymmetric contact with macrophage membranes [20,22]. Worm-like filomicelles exhibit shape- and length-dependent biodistribution; early studies demonstrated markedly prolonged circulation compared with spherical micelles, whereas subsequent studies indicate that tumor accumulation and cellular uptake depend strongly on filament length and formulation characteristics [23]. Disk-shaped and platelet-mimicking particles have attracted interest for their ability to adhere to vascular walls and avoid margination from blood flow. The implications for drug delivery design are clear, where the shape must be selected in conjunction with the intended delivery route, target tissue, and cellular uptake pathway rather than defaulting to spherical geometry. Figure 2 illustrates representative examples of non-spherical nanoparticle geometries employed in drug delivery applications, including filomicelles, bottlebrush structures, worm-like vesicles, nanorods, prisms, cylinders, disks, and ellipsoids. These morphologies exhibit distinct hydrodynamic behavior, circulation profiles, and cellular interactions, further highlighting the importance of shape as a critical parameter in the rational design of nanocarriers [24].

Surface charge, quantified by zeta potential, governs colloidal stability, protein adsorption, and cellular interaction. Cationic surfaces facilitate electrostatic interaction with the negatively charged cell membrane, improving uptake efficiency for nucleic acid delivery, but also increase non-specific protein adsorption and MPS recognition in vivo. Anionic and neutral surfaces generally offer better systemic circulation but lower intrinsic cellular internalization [25]. Surface functionalization with hydrophilic polymers such as polyethylene glycol (PEG) is commonly used to generate a ‘stealth’ coating. PEG chains form a hydrated steric barrier around the nanoparticle surface that reduces nonspecific protein adsorption and recognition by the mononuclear phagocyte system, thereby prolonging systemic circulation [14]. Liposomes are vesicular nanocarriers composed of phospholipid bilayers surrounding an aqueous core, enabling the encapsulation of therapeutic compounds within the aqueous compartment or lipid bilayer [26]. A clinically established example is PEGylated liposomal doxorubicin, in which doxorubicin is encapsulated within PEG-coated phospholipid liposomes. This formulation illustrates how surface PEGylation and liposomal encapsulation can alter drug pharmacokinetics and reduce systemic toxicity compared with free doxorubicin [27]. Drug loading efficiency depends on the physicochemical compatibility between the drug and the carrier matrix, and on whether passive (encapsulation during self-assembly) or active (post-formation loading driven by pH or ion gradients) loading strategies are employed. Active loading generally achieves higher efficiency and more controlled loading for ionizable drugs [28].

### 2.2. Pharmacokinetics, Biodistribution, and Navigating Biological Barriers

The pharmacokinetics of nanoparticle-based therapeutics differ fundamentally from those of free drugs, and understanding these differences is essential for rational dosing and the prediction of therapeutic outcomes. Following systemic administration, nanoparticles encounter a sequential series of biological barriers, each of which can attenuate the fraction of the administered dose that ultimately reaches the therapeutic target [21]. The first barrier is the blood compartment, where protein corona formation occurs within seconds of injection, altering the particle’s identity as perceived by the immune system and potentially redirecting biodistribution to MPS organs regardless of the engineered targeting strategy [15]. Stealth coatings mitigate but do not eliminate this effect, and anti-PEG antibodies generated after repeated dosing can accelerate clearance in a subset of patients, the accelerated blood clearance (ABC) phenomenon.

Vascular extravasation into solid tumors is typically achieved through the EPR effect for nanoparticles in the appropriate size range, but the magnitude of EPR-mediated accumulation is highly variable and has been called into question as a reliable basis for clinical performance [29]. Heterogeneous tumor vascularization, variable pericyte coverage, and inter-patient differences in lymphatic function mean that EPR-mediated targeting can differ by an order of magnitude across tumor types and patients, explaining in part why nanoparticle systems that show compelling efficacy in murine tumor models frequently underperform in human clinical trials [2]. Transcytosis, receptor-mediated endocytosis, and active targeting using ligands that bind disease-specific surface receptors provide more predictable targeting mechanisms that do not rely on the anatomical features of the tumor vasculature [2].

Cellular barriers constitute the final stage of delivery. Following uptake via endocytosis, nanoparticles enter the endo-lysosomal pathway, where progressively acidifying compartments (from pH 7.4 in early endosomes to pH 4.5–5.0 in lysosomes) can degrade acid-sensitive cargo and expose lysosomal nucleases that degrade unprotected nucleic acids [30]. Endosomal escape is therefore a critical design objective for the intracellular delivery of biologics and gene therapies. Ionizable lipids that undergo protonation and membrane-disruptive conformational change at low pH, proton sponge polymers that buffer acidification and induce osmotic swelling, and photosensitizer-mediated membrane disruption are among the strategies employed to achieve cytoplasmic delivery after endosomal uptake [31,32]. Subcellular targeting (delivery to the nucleus for gene therapy, to the mitochondria for metabolic interventions, or to specific organelles for precision pharmacological action) represents the next frontier in intracellular targeting and requires additional targeting sequences beyond those sufficient for cell-level selectivity [22].

## 3. Material Systems, Synthesis, and Functionalization

### 3.1. Key Nanoparticle Platforms: Polymeric, Lipid-Based, Inorganic, Carbon-Based, and Hybrid

The choice of material platform is the most fundamental decision in nanoparticle design because it determines the physicochemical properties, drug loading capacity, degradation behavior, and biological interactions of the resulting system. Table 1 provides a systematic comparison of the principal nanoparticle platforms across these dimensions. Polymeric nanoparticles (PNPs) are among the most extensively studied platforms due to their compositional versatility, tunable degradation rates, and the wide range of drugs and biologics they can encapsulate. Polyesters such as poly(lactic-co-glycolic acid) (PLGA), polylactic acid (PLA), and polycaprolactone (PCL) degrade hydrolytically to non-toxic metabolites and are the basis of the majority of clinically progressed polymeric systems [10,33]. The polymer molecular weight, copolymer composition, and end-group chemistry collectively determine the degradation rate and drug release kinetics, providing extensive tunability for sustained-release applications ranging from days to months.

**Figure 3:**
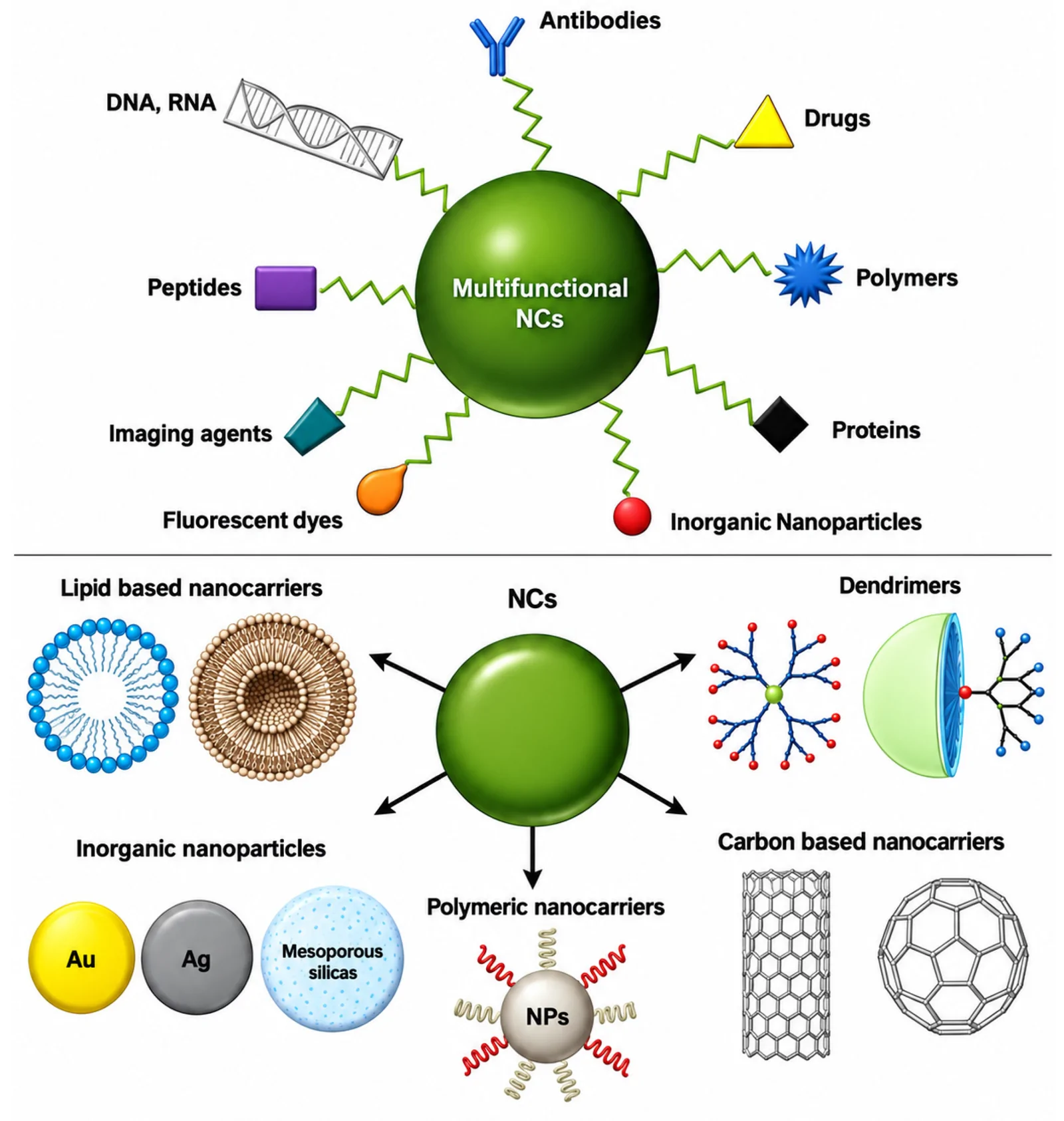
Representative nanoparticle platforms used in drug delivery and their multifunctional capabilities, including the incorporation of drugs, nucleic acids, proteins, imaging agents, and targeting ligands within a single nanocarrier system. Reprinted from Ref. [40], under the terms of the Creative Commons CC BY license.

Beyond their structural and compositional differences, modern nanoparticle platforms are increasingly designed as multifunctional systems capable of simultaneously integrating therapeutic, diagnostic, and targeting functionalities. Figure 3 illustrates representative nanocarrier architectures and the diverse range of payloads and functional moieties that can be incorporated into these systems [40].

Lipid-based nanoparticles, including liposomes and lipid nanoparticles (LNPs), represent the most clinically successful nanoparticle class to date, encompassing several FDA-approved products and the transformative COVID-19 mRNA vaccines [13,31]. Their biocompatibility, biodegradability, and the structural amphiphilicity that allows for the simultaneous encapsulation of hydrophilic cargo in the aqueous core and hydrophobic drugs in the lipid bilayer are key advantages. The development of ionizable lipids (lipids with pKa values in the range of 6–7 that are neutral at physiological pH but positively charged in acidic endosomal compartments) was a critical advance that enabled efficient siRNA and mRNA delivery by facilitating endosomal membrane disruption and cargo release [31]. Mesoporous silica nanoparticles (MSNs) offer a distinctive combination of high internal surface area (>1000 m^2^ g^−1^), tunable and uniform pore dimensions (2–50 nm), and chemical stability that makes them exceptionally capable drug reservoirs [35]. The pores can be functionalized with gating molecules (molecular machines such as nanovalves, pseudorotaxanes, and DNA duplexes) that open in response to specific stimuli, transforming the MSN into a precisely controlled drug release device [34].

Inorganic nanoparticles, including gold, iron oxide, and quantum dot systems, derive their clinical utility primarily from unique physical properties unavailable to organic carriers. Gold nanoparticles exhibit strong and size-tunable surface plasmon resonance, enabling both highly sensitive optical imaging and photothermal conversion, while superparamagnetic iron oxide nanoparticles (SPIONs) provide MRI contrast enhancement and are responsive to external magnetic fields for guided delivery and magnetic hyperthermia [36,37]. Hybrid nanoparticles that combine a polymeric core with a lipid shell (the lipid-polymer hybrid nanoparticle) represent an attempt to capture the stability and drug-loading advantages of polymers with the biocompatibility and surface versatility of lipids within a single architecture [7]. Carbon dots, a recently emerged class of photoluminescent carbon-based nanomaterials smaller than 10 nm, combine biocompatibility, tunable emission, and surface functionalizability with inherent EPR-mediated tumor accumulation, making them attractive candidates for simultaneous cancer imaging and drug/gene co-delivery [38]. Sustainable biopolymer-derived nanocarriers, including cellulose nanofibers and nanocellulose-based composites, are also emerging as promising platforms due to their biocompatibility, biodegradability, high surface area, and ease of surface functionalization for drug delivery and biomedical applications. Exosomes and red blood cell membrane-coated nanoparticles represent the biomimetic frontier, exploiting naturally derived lipid bilayers to achieve stealth circulation, and in the case of exosomes, inherent tissue targeting capabilities derived from their cell of origin [15,39].

### 3.2. Fabrication, Encapsulation Methods, and Manufacturing Scalability

The transition from materials design to a manufacturable product requires fabrication processes that reliably produce nanoparticles meeting defined critical quality attributes (CQAs), including specifications for size, polydispersity, surface charge, drug loading, and encapsulation efficiency, which are predictive of clinical performance [28]. Historically, liposomes and polymeric nanoparticles were formed by bulk mixing methods such as thin-film hydration, nanoprecipitation, and solvent emulsification, which produce acceptable laboratory-scale particles but suffer from poor process control, high batch-to-batch variability, and difficulty scaling to commercial volumes without reoptimization [28]. The introduction of microfluidic fabrication technologies represented a paradigm shift. By confining mixing to channels of defined geometry at controlled flow rates, microfluidic systems achieve rapid, reproducible nanoprecipitation with size control as fine as ±5 nm, and the laminar-to-turbulent mixing transition can be precisely tuned to optimize nanoparticle formation kinetics [41]. More recently, impingement jet mixing (IJM) technology has enabled the scale-up of LNP manufacturing to commercial volumes while preserving the mixing precision achieved by microfluidics at smaller scales, with multi-jet configurations allowing proportional increases in throughput without altering the critical hydrodynamic mixing parameters [42]. Active loading strategies (post-formation drug loading driven by transmembrane pH or ammonium sulfate gradients) achieve significantly higher encapsulation efficiencies for ionizable drugs than passive co-encapsulation during particle formation, and are the basis of the remote loading strategy used in Doxil^®^ and related pegylated liposomal products [28,43].

### 3.3. Surface Engineering: Targeting Ligands, Stealth Coatings, and Stimuli-Responsive Designs

Surface engineering is the process by which a nanoparticle with appropriate bulk properties is transformed into a precisely programmed biological agent, one that knows where to go, when to release its cargo, and how to avoid immune detection along the way. Stealth coatings are the most fundamental surface modification, and polyethylene glycol (PEG) has been the dominant strategy for more than three decades. PEGylation creates a hydrophilic, flexible polymer brush that sterically prevents protein adsorption, reduces MPS recognition, and extends circulation half-life from minutes to hours or days [14,44]. However, the clinical adoption of PEGylated systems has revealed a limitation. Repeated dosing in a subset of patients generates anti-PEG antibodies that accelerate clearance and can trigger hypersensitivity reactions. Polyzwitterionic coatings and naturally derived polymer coatings such as polysarcosine are being investigated as PEG alternatives that preserve stealth properties without immunogenic liability [45].

Active targeting is accomplished by conjugating targeting ligands (small molecules, peptides, aptamers, or antibodies) to the nanoparticle surface at densities that allow multivalent binding to disease-associated receptors while preserving the steric properties of the stealth layer. The folate receptor, transferrin receptor, ErbB2/HER2, EGFR, and PSMA are among the most intensively targeted receptors in cancer nanomedicine, while CXCR4 and VCAM-1 are studied in cardiovascular and inflammatory applications [2,46]. Folic acid-targeted carbon quantum dot systems have demonstrated dual siRNA delivery and selective cytotoxicity in folate receptor-overexpressing osteosarcoma cells, illustrating how ligand–receptor specificity can be combined with dual cargo delivery in a single platform [47]. Stimuli-responsive surface designs add a further level of programmability: pH-sensitive bonds that expose targeting ligands only in the acidic tumor microenvironment, enzyme-cleavable coatings that are stripped by tumor-associated proteases to reveal the active targeting surface, and thermo-responsive polymers that undergo conformational transitions at specified temperatures to release cargo or alter surface accessibility [10,48]. The convergence of stealth coatings, active targeting, and environmental responsiveness in hybrid surface architectures represents the current frontier of nanoparticle surface engineering.

## 4. Release Mechanisms and Advanced Delivery Strategies

### 4.1. Controlled Release via Diffusion, Degradation, and Endogenous Triggers

Controlled drug release from nanoparticles is rarely governed by a single mechanism. Rather, it typically reflects the simultaneous and coupled operation of diffusion, matrix degradation, and erosion, with their relative contributions determined by the specific polymer composition, drug physicochemical properties, and environmental conditions at the delivery site [10,49]. In biodegradable polymeric systems based on PLGA and PCL, drug molecules traverse the matrix through water-filled pores that develop as hydrolysis proceeds, and the rate of hydrolytic bond cleavage, which sets the degradation rate, is tunable through the adjustment of copolymer composition, molecular weight, and end-group chemistry [10]. As described by Kamaly et al. [10], drug release from biodegradable polymeric systems commonly results from the combined contributions of diffusion, degradation, and erosion rather than from a single mechanism. Understanding and controlling these interconnected processes through rational polymer design can enable the precise spatial and temporal regulation of drug release.

Endogenous chemical triggers exploit pathological features of disease sites to achieve selective drug release. pH-responsive systems exploit the acidic microenvironment of tumors (extracellular pH 6.5–7.0) and the even more acidic lysosomal compartment (pH 4.5–5.0) to activate acid-labile bonds or protonate ionizable lipids that then disrupt endosomal membranes, enabling cytoplasmic cargo escape [31,32]. Redox-responsive systems exploit the approximately 1000-fold difference in glutathione (GSH) concentration between the reducing cytoplasm of cancer cells (2–10 mM) and the oxidizing extracellular space (~2 µM), using disulfide crosslinks that are stable in circulation but cleaved intracellularly to trigger cargo release with high selectivity for cancerous over healthy tissue [12,50]. Enzyme-responsive designs use cleavable substrates for cathepsin B, matrix metalloproteinases (MMP-2/9), or other enzymes overexpressed in the tumor microenvironment as gatekeeping elements that remain intact in healthy tissue but are cleaved at the target site [34,51]. MSNs are particularly well-suited to enzyme-gated delivery because their pores can be sealed with supramolecular caps (pseudorotaxanes, DNA duplexes, protein gatekeepers) that are displaced or cleaved by specific biological signals, providing digital on/off control over drug release rather than the analog, diffusion-governed release of polymer systems [34]. Table 2 provides a systematic overview of the principal release mechanisms, their molecular bases, and their most relevant clinical application contexts.

### 4.2. Externally Triggered Systems: Light, Magnetic Field, and Ultrasound

Externally triggered release systems give clinicians active spatiotemporal control over drug liberation by applying a physical stimulus that is absent during circulation and storage but applied precisely to the therapeutic target. Photothermal systems based on gold nanoparticles and polymer-embedded photothermal agents leverage the surface plasmon resonance of metal nanostructures (or the strong near-infrared (NIR) absorption of organic dye-loaded polymer matrices) to convert incident light energy into localized heat with high efficiency [37,52]. In photothermal cancer therapy, this heat selectively destroys tumor cells through protein denaturation and membrane disruption while simultaneously triggering the release of thermosensitive drug payloads. Gold nanoparticles offer shape-dependent tunability of the plasmon resonance. Nanorods can be tuned to absorb maximally in the tissue-transparent NIR window (650–950 nm), nanostars exhibit a “lightning rod” concentration of electromagnetic field intensity at their sharp tips, enabling deeper tissue penetration, and nanoshells provide a structurally robust option for clinical translation [37]. For photodynamic therapy, photosensitizer-loaded nanoparticles generate reactive oxygen species upon light irradiation, providing an alternative cytotoxic mechanism that is independent of thermal effects and suitable for oxygen-replete tumor microenvironments [54].

Magnetically triggered systems use superparamagnetic iron oxide nanoparticles (SPIONs) that can be simultaneously guided to the target site by an external magnetic field and activated by an alternating magnetic field (AMF) to generate local hyperthermia through Néel and Brownian relaxation [36]. The combination of spatial guidance and thermal activation provides two independent levels of targeting control that synergize to improve tumor-to-background drug accumulation while limiting systemic exposure. Functionalization with targeting ligands and enzyme-responsive drug linkers further extends the specificity of this platform [51]. Ultrasound-mediated drug delivery exploits acoustic cavitation (the oscillation and collapse of microbubbles in an ultrasonic field) to create transient pores in cell membranes (sonoporation) that dramatically increase the uptake of co-administered nanoparticles and drugs [53]. Focused ultrasound systems allow this effect to be spatially confined to a tissue volume of millimeter-scale precision, enabling delivery through the blood–brain barrier by transient BBB opening. This approach has progressed to Phase I/II clinical evaluation, including studies in patients with recurrent glioblastoma (e.g., NCT03744026) [55].

### 4.3. Combination, Sequential, and Theranostic Delivery

The conceptual unification of diagnosis and therapy in theranostic nanoparticle systems, and the combination of multiple therapeutic modalities in a single carrier, represent logical extensions of the nanoparticle multifunctionality that distinguishes nanomedicine from conventional drug formulation. Co-delivery of cytotoxic drug combinations at defined molar ratios has demonstrated synergistic antiproliferative effects in cell culture and animal models, and the clinical success of Vyxeos^®^ (CPX-351) (a liposomal formulation of daunorubicin and cytarabine at a fixed 5:1 molar ratio) provides proof-of-concept validation that nanoparticle-enforced drug ratio maintenance can translate into meaningful survival improvements in patients [13]. Sequential release strategies deliver multiple drugs in a defined temporal sequence from the same carrier, exploiting the different sensitivity of the target cells to different treatments at different stages. Amphiphilic Janus nanoparticles with distinct hydrophilic and hydrophobic domains enable the simultaneous loading of two drugs in separate compartments and their sequential release through independent triggers (for example, pH-responsive release of one agent in the endosome followed by NIR-triggered release of the second agent in the cytoplasm), generating synergistic effects not achievable through simultaneous co-delivery [11,12].

Photo-triggered theranostic nanoparticle systems combine NIR-light-activated drug release with photoacoustic or fluorescence imaging signals generated by the same chromophore, enabling the real-time monitoring of drug distribution and tumor response during treatment [54]. MSN-based theranostic systems incorporate imaging agents (MRI contrast agents, photoacoustic dyes, or radioactive tracers) directly into the silica framework or pore channels, allowing simultaneous CT, MR, and optical imaging to guide drug delivery in real-time [11,35]. The convergence of therapeutic action and diagnostic feedback in a single injectable agent eliminates the temporal and spatial uncertainty inherent in sequentially administering separate diagnostic and therapeutic agents and represents a fundamental advance in treatment monitoring capability. Polymeric nanoparticle platforms further extend this concept through the incorporation of “smart” stimuli-responsive elements that respond to the specific biochemical environment of the tumor, enabling both activation of the therapeutic payload and the generation of a detectable signal that confirms correct localization before drug release commences [33,47].

## 5. Therapeutic Applications and Preclinical to Clinical Translation

### 5.1. Major Disease Targets: Oncology, Infectious Diseases, and CNS/CVD Disorders

Oncology has been and remains the dominant application domain for nanoparticle-based drug delivery, driven by the unmet need for treatments that can improve the therapeutic index of highly toxic chemotherapeutic agents and the growing portfolio of molecularly targeted therapies that require intracellular delivery [2,56]. Nanoparticles accumulate preferentially in tumor tissue through EPR-mediated passive targeting, and the tumor microenvironment provides multiple endogenous stimuli (acidic pH, elevated GSH, hypoxia, overexpressed proteases) for triggered drug release [12,50]. Clinical translation is exemplified by PEGylated liposomal doxorubicin, which maintains antitumor activity while reducing cardiotoxicity compared with conventional doxorubicin, demonstrating how nanoparticle encapsulation can improve the therapeutic index of established chemotherapeutic agents [57]. Carbon dots have emerged as a functionally versatile nanoplatform for simultaneous cancer imaging and drug/gene delivery, exploiting their photoluminescent properties for tumor visualization while serving as carriers for therapeutic payloads including chemotherapeutic drugs, siRNAs, and targeted gene silencing agents [38]. Folic acid-functionalized carbon quantum dot systems incorporating macrophage cell membrane coatings demonstrated the simultaneous delivery of two siRNAs targeting osteosarcoma-relevant pathways (with in vivo results showing significant tumor regression and suppression of lung metastasis without systemic toxicity), representing the integration of biomimetic stealth, active targeting, and multi-cargo delivery in a single platform [47]. Gold nanoparticles functionalized for photothermal applications have shown significant preclinical efficacy in breast cancer models, with simulation studies confirming thermal confinement within tumor tissue without damage to surrounding normal tissue [37,58].

Polysaccharide-based nanoparticle platforms have demonstrated clinically relevant performance across multiple disease targets, including cancer, cardiovascular disease, and neurodegenerative disorders, by combining biocompatibility with the chemical versatility that polysaccharides offer for surface functionalization and stimuli-responsive design [12]. In glioblastoma treatment, silica nanoparticle systems and nanoparticle-stem cell combination approaches have shown preclinical promise by protecting therapeutic payloads, enabling sustained local drug delivery and improving gene/siRNA delivery within the immunosuppressive brain tumor microenvironment [32]. For cardiovascular applications, nanoparticle-based delivery of anti-inflammatory and antifibrotic agents to sites of vascular injury and myocardial infarction exploits the selectivity of targeted nanocarriers for activated endothelium and macrophage-rich plaques, with enzyme-responsive magnetic nanoparticles combining MRI-guided localization with spatially precise drug activation [51]. Clinical translation of CNS delivery is also being explored using focused ultrasound-mediated blood–brain barrier opening. Early-phase clinical evaluation, including a Phase I/II study in recurrent glioblastoma (NCT03744026), has demonstrated the feasibility of transient BBB opening to facilitate therapeutic delivery [55]. Infectious disease applications include both direct antimicrobial delivery, where nanoparticle-encapsulated antibiotics achieve sustained local concentrations at infection sites, and vaccine delivery, where nanoparticles serve as antigen carriers and adjuvant platforms that improve immunogenicity and reduce dosing frequency [59,60].

### 5.2. Gene Delivery and Vaccines

Gene therapy and RNA interference represent therapeutic modalities that fundamentally require nanoparticle delivery due to the inherent pharmacological limitations of nucleic acids as free agents. They are rapidly degraded by serum nucleases, do not cross cell membranes passively due to their large size and negative charge, and in the case of plasmid DNA, must reach the nucleus for transcription [30]. Lipid nanoparticles with ionizable lipid components are the leading platform for nucleic acid delivery, exemplified by the clinical success of patisiran, the first FDA-approved siRNA therapeutic, which uses LNPs to achieve efficient hepatocyte-targeted gene silencing in hereditary transthyretin amyloidosis, and by the COVID-19 mRNA vaccines, which established the largest clinical validation of LNP-mediated nucleic acid delivery in history [8,31]. In the pivotal Phase III APOLLO trial, patisiran significantly improved polyneuropathy and quality-of-life measures compared with the placebo, providing direct clinical validation of systemic LNP-mediated siRNA delivery [61]. Similarly, pivotal Phase III trials of LNP-formulated mRNA COVID-19 vaccines demonstrated 95.0% efficacy for BNT162b2 and 94.1% efficacy for mRNA-1273 against symptomatic COVID-19, providing large-scale clinical validation of LNP-mediated mRNA delivery [62,63,64]. The key mechanistic challenge addressed by ionizable LNPs is endosomal escape. At lysosomal pH, the ionizable lipid becomes protonated, assumes a positive charge, and disrupts the endosomal membrane through charge-driven lipid mixing, releasing siRNA or mRNA into the cytoplasm, where it can access the RNA interference machinery or ribosomes [31].

Carbon dots and polymeric carriers have demonstrated capacity for multi-cargo nucleic acid delivery in preclinical cancer models, with folic acid-targeted platforms simultaneously delivering two siRNAs targeting complementary oncogenic pathways at the same tumor cell, demonstrating that nucleic acid cargo multiplicity is achievable without compromising targeting or cellular uptake efficiency [47]. Exosome-based delivery systems represent a biomimetic alternative to synthetic nucleic acid carriers that exploits the natural intracellular communication function of exosomes. Their inherent membrane stability, natural targeting capabilities encoded by surface proteins from their cell of origin, and reduced immunogenicity relative to synthetic liposomes are advantageous for gene therapy applications where repeated dosing may be required [39]. Vaccine delivery using nanoparticles extends beyond mRNA vaccines to encompass protein antigen delivery with polysaccharide-based systems that achieve sustained antigen release over three or more weeks, enabling single-dose immunization protocols that are particularly valuable for programs targeting vaccine-hesitant or geographically dispersed populations [20,59]. Hydrogel nanoparticle systems for intranasal vaccine delivery have demonstrated improved mucosal immune responses in animal models compared to conventional intranasal formulations, attributable to the improved mucoadhesion and prolonged antigen residence time at the nasal-associated lymphoid tissue [59].

### 5.3. Evaluation Pipeline: In Vitro Models, In Vivo Studies, and Clinical Trials

The evaluation pipeline for nanoparticle drug delivery systems follows a progression from in vitro cellular models through in vivo preclinical studies to clinical trials, each stage providing increasingly complex and predictive biological data at progressively higher cost and regulatory scrutiny. In vitro studies provide initial evidence of therapeutic activity, cellular uptake, and cytotoxicity selectivity at low cost and high throughput, with two-dimensional cell cultures being supplemented by more predictive three-dimensional tumor spheroids, organoids, and organ-on-chip systems that better replicate the spatial heterogeneity and mass transport barriers of solid tumors [47,65]. Assessment of in vitro drug release kinetics under physiologically relevant conditions (including buffer pH, enzyme concentration, and temperature matching the intended delivery environment) provides a mechanistic understanding of release behavior that informs in vivo dose prediction and study design. For folic acid-targeted carbon quantum dot systems, in vitro studies demonstrated the inhibition of osteosarcoma cell proliferation and invasion, dose-dependent cytotoxicity, and pH-responsive drug release profiles that provided the mechanistic rationale for in vivo testing [47].

In vivo studies in rodent xenograft and syngeneic tumor models provide evidence for systemic pharmacokinetics, biodistribution, tumor accumulation efficiency, and therapeutic efficacy in a living organism with intact vasculature and partially functional immune response [65]. Significant tumor regression and the suppression of distant metastasis without systemic toxicity in in vivo models represent the evidence threshold typically required before clinical investigation begins, and has been demonstrated for multiple carbon dot, polysaccharide, and MSN-based systems in preclinical oncology work [47,59]. However, the predictive value of murine preclinical data for human clinical outcomes has been consistently questioned, particularly for tumor targeting. This limitation has been demonstrated experimentally for lipid nanoparticle (LNP)-mediated mRNA delivery. In a comparative study evaluating 89 chemically distinct LNPs across murine, non-human-primate-derived, and human hepatocytes in vivo, delivery to non-human primate and human hepatocytes showed a strong correlation (R^2^ = 0.83), whereas the correlation between murine and human hepatocytes was substantially lower (R^2^ = 0.53). These findings demonstrate that nanoparticle delivery performance can be species-dependent and highlight the importance of selecting physiologically relevant preclinical models before clinical translation [66]. The EPR effect is more pronounced and reproducible in subcutaneous xenograft models than in orthotopic, spontaneous, or patient-derived xenograft (PDX) models that better reflect the vascular and immunological complexity of human tumors [29]. This disconnect between preclinical promise and clinical performance is reflected in the substantial attrition of nanoparticle-based oncology therapeutics during clinical development, highlighting the limited predictive value of many conventional preclinical models for human outcomes [13]. Approved nanoparticle products such as Doxil^®^, Abraxane^®^, Onpattro^®^, and the mRNA vaccines confirm that clinical success is achievable, but that the pathway from compelling preclinical data to approved therapeutic is long, expensive, and uncertain [13]. Table 3 provides an overview of key approved and late-stage clinical nanoparticle products.

## 6. Computational Design, Artificial Intelligence, and Machine Learning

### 6.1. AI-Driven Design of Nanoparticle Formulations and Properties

The integration of artificial intelligence and machine learning into nanoparticle design represents a paradigm shift from the iterative empirical approach, namely synthesize, characterize, and modify, that has governed the field since its inception. The fundamental limitation of empirical optimization is that nanoparticle performance depends on the simultaneous interaction of dozens of formulation variables (lipid composition, molar ratios, polymer molecular weight, surface ligand density, drug loading method) and process variables (mixing conditions, temperature, flow rate), creating a parameter space too vast for exhaustive experimental exploration [3,67]. Machine learning models trained on existing experimental datasets can identify multivariate structure–property relationships across this space and predict optimal formulation orders of magnitude faster than laboratory synthesis, making them invaluable for initial formulation screening before physical experiments are undertaken.

Tunable Nanoparticles using Artificial Intelligence (TuNa-AI) is a hybrid machine-learning framework developed to guide nanoparticle formulation by learning relationships between formulation composition and experimentally measured performance. The approach integrates formulation data with predictive modeling to identify optimized nanoparticle compositions while reducing unnecessary formulation components and experimental iterations [67]. Notably, in a trametinib formulation, the AI-guided approach reduced excipient usage by 75% while preserving in vitro efficacy and in vivo pharmacokinetics relative to the standard formulation, demonstrating the potential of machine learning to simplify nanoparticle compositions while maintaining therapeutic performance [67]. The development of science-specific language models such as LightGBM for gradient-boosted decision tree learning, combined with microfluidic synthesis platforms that generate high-quality training data rapidly and reproducibly, is creating a closed-loop computational-experimental design cycle in which AI predictions are tested, results are fed back as new training data, and model accuracy improves iteratively [41,67]. For mRNA-LNP manufacturing, AI-coupled microfluidic systems have been demonstrated to achieve the precise control of particle size and morphology, parameters that critically influence mRNA delivery efficiency and immunogenicity, within a quality-by-design framework that is directly compatible with pharmaceutical manufacturing standards [41,42].

### 6.2. Modeling Drug Release, Pharmacokinetics, and Targeting Efficacy

Computational pharmacokinetic modeling occupies a critical role in the nanoparticle development pipeline by providing quantitative predictions of in vivo behavior that inform dose selection, schedule optimization, and the design of clinical studies before first-in-human testing. Multi-view learning neural networks trained on physicochemical nanoparticle descriptors and animal pharmacokinetic data have been demonstrated to predict key PK parameters (area under the curve (AUC), maximum plasma concentration, and clearance rate) across multiple nanoparticle classes with predictive accuracy sufficient for dose range selection in preclinical studies [68]. These predictive models accelerate the transition from formulation design to in vivo testing by reducing the number of pharmacokinetic studies required to characterize a new formulation, and their integration with population PK modeling approaches is enabling patient-specific dose personalization based on individual physiological parameters [68,69].

AI-based protein design tools, including RFdiffusion, enable de novo generation of proteins with specified structural and functional properties and may provide a foundation for the rational development of targeting ligands for nanoparticle delivery systems [70]. The ANIDS AI system has been proposed as a computational framework for optimizing nanoparticle formulations and illustrates the potential of AI-guided approaches for simultaneously considering targeting and drug-encapsulation parameters [22]. However, independent experimental validation across diverse nanoparticle systems is still required before the predictive performance and generalizability of such approaches can be established. Table 4 summarizes the principal AI and machine learning approaches being applied to nanoparticle research, their specific applications, and the key outcomes demonstrated.

### 6.3. Predictive Toxicology and Personalized Nanomedicine

The application of AI to predictive nanotoxicology addresses one of the most time-consuming and resource-intensive aspects of nanoparticle development such as the characterization of safety profiles for materials with large compositional spaces. Traditional nanotoxicology testing evaluates one variable at a time (nanoparticle size, then surface charge, then composition, in sequence), generating incomplete safety pictures that cannot capture the interaction effects between multiple variables that commonly govern real biological responses [71]. Random forest ensemble models trained on multi-dimensional nanotoxicology datasets can simultaneously evaluate the size, surface chemistry, shape, and composition as predictors of cytotoxicity, genotoxicity, and inflammatory response, identifying non-obvious safety signals that univariate testing would miss while dramatically reducing the animal experiment burden required to establish preliminary safety profiles [71]. These predictive toxicology tools are now being integrated into the early formulation design process, enabling the identification of potentially toxic compositions before laboratory synthesis, a true safety-by-design paradigm for nanomedicine.

Recent studies provide practical examples of this potential. TuNa-AI integrates automated high-throughput nanoparticle synthesis with machine learning to optimize both material selection and formulation composition. Using a dataset of 1275 formulations, the platform increased successful nanoparticle formation by 42.9% through composition optimization, and in a trametinib case study, reduced excipient use by 75% while maintaining in vitro efficacy and in vivo pharmacokinetics [67]. Such approaches illustrate how AI can move beyond retrospective data analysis toward closed-loop formulation design, in which computational predictions guide experimental synthesis and validation. Looking forward, integration with pharmacokinetic modeling and patient-specific digital twins could further support individualized formulation selection and dosing, although these applications still require substantial clinical validation.

The most transformative near-term prospect for AI in nanomedicine is the development of truly personalized nanoparticle therapeutics in which drug formulation, dose, and administration schedule are individually optimized for each patient based on their genomic, proteomic, and clinical data [13,73]. In silico digital twin (IST) frameworks implement this concept by creating a patient-specific computational replica (parameterized with the individual’s age, genetic polymorphisms, organ function indices, and disease characteristics) that simulates the pharmacokinetics, tissue distribution, and pharmacodynamics of candidate nanoparticle formulations before any drug is administered [72]. By running thousands of simulated dosing scenarios on the patient’s digital twin, the optimal formulation and schedule can be identified, and predicted adverse effects can be anticipated and mitigated before clinical exposure. This approach is particularly valuable for patient populations with variable metabolism (pediatric, elderly, renally impaired, and genetically polymorphic individuals) who are systematically underrepresented in the clinical trials that inform standard dosing recommendations [72].

## 7. Safety, Regulation, and Societal Impact

### 7.1. Biocompatibility, Long-Term Toxicity, and Immunogenicity

The assessment of nanoparticle biocompatibility demands a more comprehensive evaluation framework than is applied to conventional small-molecule drugs because nanoparticles interact with biological systems as physical objects through their geometry, surface area, and charge, in addition to their chemical properties, and because their behavior at the cellular and subcellular levels cannot be predicted from bulk material properties alone [17]. Cytotoxicity assessment in relevant cell lines provides an initial indication of cellular safety, but must be distinguished from systemic biocompatibility in multi-tissue organisms, where compensatory mechanisms, organ-level accumulation, and chronic exposure effects introduce complexities absent from cell culture models [17]. Robust safety evaluation therefore requires in vitro panels covering hepatic, renal, pulmonary, and immune cells, followed by in vivo studies assessing systemic toxicity, genotoxicity, carcinogenicity, and reproductive toxicity through standard toxicological protocols adapted for nanoparticle-specific considerations [17].

The immunogenicity of nanoparticle systems presents a particularly complex safety challenge because the immune system responds to nanoparticles through multiple overlapping mechanisms (complement activation, macrophage phagocytosis, T-cell activation, and antibody-mediated recognition), and these responses can be either beneficial (adjuvant effects in vaccines) or detrimental (inflammatory reactions, anti-drug antibody formation, accelerated blood clearance), depending on the specific nanoparticle composition and application [18]. The formation of anti-PEG antibodies following repeated dosing with PEGylated nanoparticles is a clinically established example of immunogenicity-driven pharmacokinetic variability that was not predicted by preclinical studies [15]. Long-term organ accumulation represents a critical safety concern for inorganic nanoparticles and carbon-based systems that are not fully biodegradable. The liver and spleen are primary sites of nanoparticle sequestration by resident macrophages, and the long-term consequences of progressive nanoparticle accumulation, including their degradation products’ effects on hepatic and splenic function, require extensive longitudinal study before clinical approval [16]. As Kutner et al. [16] have emphasized, even biodegradable nanoparticle systems depend on the safety of their degradation products, since accumulated byproducts may themselves provoke inflammation or foreign body responses distinct from those caused by the intact carrier.

### 7.2. Regulatory Frameworks and Standardization Pathways

The regulatory classification of nanomedicine products is a fundamental determinant of the development pathway, approval timeline, and post-market surveillance requirements a product faces. The U.S. FDA, the European Medicines Agency (EMA), and the Japanese PMDA classify nanoproducts as drugs, biologics, or medical devices based on their primary mode of action, with each classification imposing different Chemistry, Manufacturing, and Controls (CMC) requirements, clinical trial designs, and safety evidence thresholds [17,18]. The complexity of nanoparticle systems (which can simultaneously exhibit drug-like pharmacological activity, device-like physical function, and biologic-like immune modulation) creates regulatory ambiguity that has been a persistent source of friction in the approval process. Nucleic acid nanoparticles represent the most acute example of regulatory boundary cases. The traditional pharmaceutical regulatory framework distinguishes between active pharmaceutical ingredients (APIs) and excipients, but a nucleic acid nanoparticle in which the lipid carrier is both the therapeutic enabler and the structural component challenges this binary classification in ways that require entirely new regulatory thinking [74].

The urgent need for standardized characterization and testing protocols for nanomedicines is recognized by regulatory agencies, standards organizations, and the academic community alike. Without agreed standards for measuring nanoparticle size distribution, surface charge, protein corona composition, and drug release profiles under physiologically relevant conditions, it is impossible to make meaningful cross-study comparisons, establish the material characterization requirements that a regulatory submission must meet, or define the performance specifications that a generic nanoparticle product must satisfy to demonstrate equivalence to an innovator product [15,17]. Organizations including ISO, ASTM, and the OECD are actively developing nanoparticle-specific standards and test guidelines to fill this gap, and the FDA and EMA have published nanotechnology-specific guidance documents that provide important initial frameworks, though global harmonization remains incomplete [17].

### 7.3. Ethical, Societal, and Cost-Accessibility Considerations

The integration of nanotechnology into clinical medicine raises ethical dimensions that extend beyond conventional pharmaceutical ethics to encompass questions specific to the novel capabilities and uncertainties of nanoscale systems. Informed consent for clinical nanoparticle trials, and eventually, approved nanoparticle therapies, require that patients receive meaningful information about nanoparticle compositions, potential long-term biodistribution, the possibility of multi-generational biological effects from persistent non-biodegradable materials, and the inherent uncertainty surrounding safety profiles that have not yet been characterized over full human lifetimes [17]. The asymmetry between the sophisticated technical understanding required to evaluate nanoparticle safety data and the lay understanding available to most patients creates a consent quality gap that is larger for nanomedicines than for conventional drugs.

Accessibility and cost equity arguably present the most consequential societal dimension of nanomedicine. The development and clinical translation of nanoparticle therapeutics can involve substantial costs associated with formulation optimization, scale-up, physicochemical characterization, preclinical evaluation, regulatory compliance, and clinical testing. These requirements may ultimately contribute to high product costs and create accessibility challenges, particularly for patients in low- and middle-income countries [75]. This economic structure risks creating a “two-tier medicine” in which the most powerful therapeutic tools available are preferentially accessible to the wealthy, widening rather than closing global health inequities. Extended-release and long-acting drug delivery formulations may improve treatment adherence by reducing dosing frequency and simplifying therapeutic regimens [75]. Sustainable manufacturing approaches using biopolymer-based and green chemistry synthesis routes directly address the cost and environmental burden dimensions of this equity challenge.

## 8. Challenges and Critical Barriers to Translation

Despite extensive preclinical research demonstrating nanoparticle efficacy across multiple disease areas, the vast majority of nanoparticle systems that show compelling laboratory performance do not reach clinical approval, reflecting a complex set of scientific, manufacturing, regulatory, and biological barriers that are distinct from those confronting conventional small-molecule drug development. Table 5 provides a structured overview of the principal barriers, their root causes, current mitigation strategies, and development status.

### 8.1. Reproducibility, Scalability, and Manufacturing Hurdles

Reproducibility at the batch level (the ability to produce nanoparticles with consistent size, loading, surface properties, and biological activity across independent manufacturing runs) is a fundamental requirement for clinical use that many laboratory-scale nanoparticle synthesis methods cannot reliably meet [28]. The sensitivity of nanoparticle formation to mixing conditions, reagent lot-to-lot variability, and minor process parameter deviations means that a synthesis protocol that produces excellent material in one laboratory may yield unacceptably variable material in another, or in the same laboratory at different times [77]. This reproducibility gap is not merely a regulatory inconvenience, it is a scientific problem that limits the ability to draw meaningful conclusions from clinical trial data when the nanoparticle formulation itself may vary between patients or even between treatment cycles within the same patient. The adoption of microfluidic and IJM manufacturing approaches with in-process analytical monitoring, combined with design of experiments (DoE) frameworks that systematically map the design space and identify process parameters critical to product quality, represents the current industrial approach to resolving this challenge [28,42].

Scalability from laboratory to commercial manufacturing is a distinct and equally challenging problem because the physical processes that govern nanoparticle formation (fluid mixing, interfacial energy minimization, nucleation and growth kinetics) do not scale linearly with volume. Increasing batch size without changing mixing conditions alters the effective mixing time and shear rate that the formulation experiences, which can change particle size distribution, drug loading, and surface properties in ways that require complete reoptimization of the manufacturing process at each scale step [41]. Green-synthesized nanoparticles, which use biologically derived reducing agents and natural extract-mediated synthesis routes, offer a potentially more environmentally sustainable manufacturing pathway, but currently lack the process control and characterization data required for regulatory filing, representing an important research agenda for sustainable nanomedicine [78,79].

### 8.2. Biological Complexity, Patient Variability, and the EPR Debate

The enhanced permeability and retention (EPR) effect has served as the foundational rationale for passive nanoparticle targeting in oncology, and it underlies the design logic of virtually every passively targeted nanoparticle oncology product [3]. However, a growing body of clinical and meta-analytic evidence has called into question the reliability of EPR-mediated tumor accumulation as a predictable clinical phenomenon. A widely cited meta-analysis by Wilhelm et al. reported that the median delivery of systemically administered nanoparticles to solid tumors was approximately 0.7% of the injected dose, highlighting the substantial biological barriers that limit nanoparticle tumor accumulation. However, subsequent analyses have emphasized that estimates of nanoparticle delivery efficiency depend on the pharmacokinetic metrics and analytical approaches used [80]. The factors governing this variability include tumor vascular density and permeability, pericyte coverage and vessel maturation, interstitial fluid pressure, and lymphatic drainage function, all of which differ substantially between the transplanted subcutaneous xenografts used in most preclinical studies and the spontaneous, stromal-rich tumors encountered in clinical oncology. This EPR controversy does not negate the value of nanoparticle delivery (the improved tolerability and pharmacokinetics of Doxil^®^ relative to free doxorubicin are real clinical benefits regardless of whether EPR is the mechanism). Still, it fundamentally undermines the assumption that passive targeting alone is sufficient for clinical efficacy and strengthens the case for active targeting strategies.

### 8.3. Standardization and Long-Term Safety Data

The absence of standardized characterization, testing, and reporting frameworks for nanoparticle drug delivery is a structural barrier that impedes progress at every stage from basic research to regulatory approval. Without agreed protocols for measuring nanoparticle size distribution by appropriate techniques (DLS, NTA, AF4-MALS), reporting protein corona composition under physiologically relevant conditions, and characterizing drug release under validated in vitro conditions that correlate with in vivo performance, the results of different research groups studying apparently similar systems cannot be meaningfully compared or synthesized into collective knowledge [15,17]. This fragmentation means that the global community of nanoparticle researchers is, to a significant degree, producing data that cannot be aggregated into the large, high-quality datasets needed to train the machine learning models and establish the mechanistic understanding required for reliable clinical translation prediction.

Long-term safety data gaps are particularly acute for nanoparticle systems containing non-biodegradable materials (gold, silica, iron oxide) that may persist in organs for months to years after administration. Preclinical toxicology studies conducted over weeks or months cannot capture the effects of years of organ accumulation that will occur in patients who receive repeated treatment cycles for chronic conditions. Similarly, the emergence of anti-PEG immune responses in clinical populations (not predicted by preclinical studies) illustrates that nanoparticle-specific immunological effects may only become apparent after years of clinical use. The nanotoxicology community, in partnership with regulatory agencies and ISO/OECD standards bodies, is actively developing standardized in vitro and in vivo test batteries specifically designed for nanoparticle safety assessment, and the establishment of these standards is recognized as a prerequisite for the accelerated development of the next generation of nanomedicines [17,18].

## 9. Emerging Trends and Future Directions

### 9.1. Personalized and Biomimetic Nanomedicine

The convergence of personalized medicine and nanomedicine is producing a new generation of nanoparticle therapeutics whose composition, surface functionalization, and dosing are tailored to the molecular and physiological profile of individual patients. AI-powered in silico digital twin frameworks represent the most comprehensive implementation of this concept, enabling patient-specific simulation of nanoparticle pharmacokinetics and pharmacodynamics before clinical exposure and the optimization of formulation and dosing schedules for each individual [72]. The clinical utility of this approach is greatest for patient populations with high inter-individual variability in drug metabolism and tissue distribution, including pediatric, elderly, and genetically polymorphic populations, where standard population-average dosing is least reliable. Personalized surface functionalization, where targeting ligands are selected to match the specific receptor expression profile of a patient’s tumor as determined by biopsy proteomics or liquid biopsy, represents a related frontier that is becoming technically feasible as next-generation protein design tools mature [46].

Biomimetic nanoparticle strategies exploit biological structures and materials (cell membranes, extracellular vesicles, protein cages, and naturally derived biopolymers) to construct delivery systems with inherent biological compatibility and targeting capabilities that synthetic materials cannot replicate. Red blood cell membrane-coated nanoparticles acquire the self-recognition “CD47” and “do not eat me” signals of erythrocytes, enabling immune evasion and prolonged circulation without PEGylation, while exosomes derived from tumor-homing immune cells, such as macrophages and NK cells, carry natural targeting ligands for tumor-associated antigens [15,39]. Wound healing and tissue regeneration applications have recently exploited nanocellulose-based scaffolds and nanomaterial-functionalized wound dressings that modulate inflammation, provide mechanical support for tissue reconstruction, and deliver therapeutic factors with spatial precision unavailable to conventional wound care formulations. These biomimetic platforms illustrate the broadening scope of nanomedicine beyond drug delivery into tissue engineering and regenerative medicine.

### 9.2. Sustainable Nanomanufacturing and Green Chemistry Approaches

The environmental footprint of nanoparticle synthesis is an increasingly important consideration as nanomedicine moves from small-scale laboratory production to industrial manufacturing. Conventional nanoparticle synthesis routes frequently employ non-biodegradable polymers, toxic organic solvents, and hazardous reducing agents whose waste streams require specialized treatment and generate significant environmental burden [78,79]. Green chemistry approaches for drug-delivery nanoparticles seek to replace hazardous solvents and reagents with safer alternatives and adopt resource-efficient manufacturing processes while preserving the physicochemical properties, biocompatibility, drug-loading capacity, and reproducibility required for therapeutic use. Table 6 summarizes representative sustainable manufacturing strategies directly relevant to nanoparticle drug-delivery systems.

For drug-delivery applications, green nanomanufacturing should focus not only on reducing environmental impact but also on preserving the physicochemical properties, biocompatibility, drug-loading capability, and reproducibility required for therapeutic use. Recent studies demonstrate that greener solvent systems can be employed for PLGA nanoparticle production while maintaining particle characteristics and cellular uptake comparable to conventional formulations [81]. Process-intensified approaches such as microfluidic synthesis may further reduce reagent and energy consumption while improving control over nanoparticle size and reproducibility [83]. Nevertheless, regulatory translation requires the rigorous characterization of residual solvents, batch consistency, stability, and biological performance before such sustainable manufacturing strategies can be adopted clinically.

### 9.3. Next-Generation Clinical Translation and Global Health Integration

The most impactful near-term opportunity for expanding nanomedicine’s global health contribution lies in closing the substantial translational gap between the large number of nanoparticle systems reported in preclinical research and the comparatively limited number that have progressed to clinical approval [13]. The primary causes of this translational attrition, poor predictive validity of standard murine models, EPR effect variability in human tumors, unresolved long-term safety concerns, and manufacturing scalability barriers, are well-characterized, and targeted research investments addressing each barrier are likely to yield proportionate improvements in clinical trial success rates. Patient-derived tumor models, including organoids and humanized mouse models with reconstituted human immune systems, provide more predictive platforms for efficacy and safety assessment that may improve the pre-clinical-to-clinical translation rate [32]. Standardized regulatory pathways with pre-competitive collaborative development of characterization standards (modeled on the ICH guidelines that have harmonized small-molecule drug development across major regulatory jurisdictions) would reduce the per-product regulatory burden and accelerate approval timelines for nanoparticle therapeutics globally.

Nanotechnology education in medical training programs represents an underappreciated systemic barrier to clinical translation. Medical practitioners who will prescribe, administer, and monitor nanoparticle-based therapeutics must understand the fundamental properties of these systems well enough to select appropriate patients, recognize unique adverse effects, and interpret performance deviations. Díaz-López et al. [84] documented significant gaps in nanotechnology awareness among postgraduate medical students and identified structural curricula limitations, including limited dedicated course time and the insufficient integration of nanotechnology into existing pharmacology and clinical oncology training, as root causes that require systemic educational reform rather than individual study. Addressing this knowledge gap is as important for clinical translation as the technical challenges, because therapeutic benefit can only be realized if clinicians possess the understanding needed to use nanomedicines correctly and safely.

## 10. Conclusions and Outlook

Nanoparticle-based drug delivery systems have moved from concept to clinical standard over the past three decades, with FDA-approved products spanning liposomal chemotherapeutics, albumin-bound nanoparticles, siRNA-LNP gene therapy, and mRNA vaccines that have collectively benefited hundreds of millions of patients. The fundamental scientific principles are well-established. The size, shape, surface charge, and composition jointly determine pharmacokinetic behavior and cellular fate; the EPR effect enables passive tumor accumulation, though with high patient-to-patient variability; stimuli-responsive mechanisms can achieve controlled, site-specific drug release with spatial and temporal precision; and multifunctional architectures integrating drug delivery with imaging and diagnostic functionality are now technically feasible across multiple material platforms.

The central challenge of the present moment is not discovery; enough nanoparticle platforms, surface engineering strategies, and targeting approaches have been characterized to fill an enormous clinical portfolio, but translation: reliably converting compelling laboratory demonstrations into manufacturable, safe, and clinically effective products that reach patients at an accessible cost. This translation challenge has multiple interacting dimensions. Manufacturing scalability requires engineering solutions that maintain nanoparticle quality attributes during commercial-scale production. Biological predictability requires improved preclinical models that faithfully replicate the immunological, vascular, and cellular complexity of human disease. Regulatory efficiency requires harmonized characterization standards and approval pathways designed for the unique properties of nanoparticle systems. Clinical utility requires patient-specific approaches that account for the inter-individual biological variability that causes population-average nanoparticle performance to diverge from the performance needed for each patient.

The emerging integration of artificial intelligence, machine learning, and digital twin frameworks into nanomedicine design is the most powerful accelerant currently available for addressing these challenges. By enabling multi-variable formulation optimization, predictive pharmacokinetics, high-throughput toxicity screening, and patient-specific treatment planning, AI tools compress the empirical iteration cycles that currently consume most of the time and cost of nanomedicine development. Sustainable green chemistry approaches address the cost and environmental barriers that limit the accessibility of nanomedicine in low-resource settings. Biomimetic platforms that exploit natural biological components for immune evasion and targeting address the immunogenicity and protein corona challenges that limit the in vivo performance of synthetic carriers. Together, these converging advances suggest that the third decade of clinical nanomedicine will be defined not by the technical achievement of ultralow friction or single-particle drug delivery precision, but by the achievement of manufacturing reliability, clinical predictability, regulatory clarity, and global accessibility that transforms nanoparticle platforms from specialized research tools into routine elements of personalized medical care.

## Figures and Tables

**Table 1:** Comparative overview of the principal nanoparticle drug delivery platforms: composition, size range, key advantages, primary limitations, and representative therapeutic applications.

Platform	Core Composition	Size Range	Key Advantages	Primary Limitations	Representative Applications	Ref.
Polymeric NPs	PLGA, PCL, PLA, chitosan, albumin	50–500 nm	Tunable degradation rate; high drug-loading capacity; surface versatility; stimuli-responsive variants available	Batch-to-batch variability; potential immunogenicity of degradation products; scale-up challenges	Oncology (paclitaxel, doxorubicin delivery); gene therapy; arthritis; CNS disorders	[7,10,33]
Lipid-based NPs (liposomes, LNPs)	Phospholipids, cholesterol, ionizable lipids	50–200 nm	Excellent biocompatibility; biodegradable; amphiphilic bilayer accommodates both hydrophilic and hydrophobic cargo; PEGylation extends circulation	Physical instability (aggregation, leakage); cold-chain storage requirements; hypersensitivity reactions in some patients	mRNA COVID-19 vaccines (Pfizer/Moderna); Doxil^®^ (oncology); siRNA delivery (Onpattro^®^)	[7,31]
Mesoporous silica NPs (MSNs)	Amorphous SiO_2_ with ordered pore channels	50–300 nm	High surface area and pore volume; tunable pore size; thermally and chemically stable; excellent drug-loading capacity; gatable pores for controlled release	Non-biodegradable in certain formulations; potential for long-term silica accumulation; synthesis complexity	Theranostics; pH/enzyme-responsive delivery; antifungal; anticancer drug loading	[34,35]
Inorganic NPs (gold, iron oxide)	Au, Fe_3_O_4_/Fe_2_O_3_, QDs, MnO_2_	5–100 nm	Unique optical/magnetic properties; excellent imaging contrast; photothermal conversion efficiency; precise targeting under external field guidance	Potential long-term accumulation; regulatory uncertainty; cost; limited biodegradability of metallic cores	Photothermal cancer therapy; MRI contrast agents; magnetic hyperthermia; colorectal cancer biosensors	[36,37]
Hybrid (lipid-polymer) NPs	polymeric core + lipid shell	50–250 nm	Combines polymer stability with lipid biocompatibility; stealth coating; co-delivery of hydrophilic and hydrophobic drugs; reduced protein corona formation	More complex fabrication; additional quality control requirements; cost of manufacturing	Combination cancer chemotherapy; boron neutron capture therapy (BSH-LDH); targeted oncology	[7]
Carbon-based NPs (carbon dots, CNTs)	sp^2^/sp^3^ carbon; surface-functionalizable	1–10 nm (CDs)	Tunable photoluminescence; biocompatible surface chemistry; EPR-mediated tumor accumulation; dual drug and gene delivery	Heterogeneous size distribution; uncertain long-term toxicity; scale-up and clinical translation barriers	Cancer theranostics; gene silencing (siRNA); tumor microenvironment-responsive imaging	[38]
Exosomes/biomimetic NPs	Cell-derived lipid vesicles; RBC membrane coatings	30–150 nm	Inherent immune evasion; natural targeting ability; low immunogenicity; capable of carrying proteins, nucleic acids, and small molecules	Complex and low-yield isolation; batch variability; challenges in large-scale production and standardization	siRNA delivery; immune modulation; CNS targeting; cancer immunotherapy	[15,39]

NPs = nanoparticles; LNPs = lipid nanoparticles; MSNs = mesoporous silica nanoparticles; CDs = carbon dots; RBC = red blood cell; EPR = enhanced permeability and retention.

**Table 2:** Systematic overview of nanoparticle drug release mechanisms: trigger type, representative carrier systems, molecular basis, and primary clinical application context.

Release Mechanism	Trigger	Nanoparticle System	Molecular Basis	Key Application Context	Ref.
Diffusion-controlled	Passive (concentration gradient)	Polymeric NPs, lipid vesicles	Drug diffuses through water-filled pores or polymer matrix without structural breakdown; rate governed by matrix permeability	Sustained systemic release; chronic disease management (hypertension, diabetes)	[9,10]
Hydrolytic degradation	Passive (aqueous environment)	PLGA, PLA, PCL nanoparticles	Ester bond hydrolysis in polyester backbone progressively fragments matrix; rate tunable via molecular weight and copolymer ratio	Long-term oncology; post-surgical local delivery; gene therapy depots	[10,49]
pH-responsive	Endosomal acidification (pH 5–6); tumor microenvironment (pH 6.5–7.0)	Ionizable LNPs; acid-labile polymers; MSN nanovalves	Ionizable lipids acquire positive charge at low pH, disrupting endosomal membrane; acid-labile linkers cleave to release cargo	Intracellular drug/siRNA delivery; cancer targeting; lysosomal escape for gene therapy	[31,32,34]
Redox-responsive	Elevated intracellular GSH (tumor cytoplasm ~10 mM vs. ~2 µM extracellular)	Disulfide-crosslinked polymeric NPs; thiolated MSNs	GSH cleaves disulfide bonds holding polymer crosslinks or gating agents, releasing encapsulated cargo selectively in reductive intracellular compartments	Cytoplasmic drug delivery; cancer-selective release; siRNA release in tumor cells	[12,50]
Enzyme-responsive	Cathepsin B, MMP-2/9 (overexpressed in tumors)	Peptide-capped MSNs; enzyme-cleavable polymer prodrugs; magnetic NPs	Enzyme cleaves substrate linker or gating peptide to open pore or activate drug; exploits tumor-associated overexpression	Tumor-selective delivery; personalized cancer therapy; precision oncology	[34,51]
Photothermal/photo-triggered	NIR light (650–950 nm); UV	Au NPs, polymer-based NPs, azobenzene-gated MSNs	Light absorbed by plasmonic or photosensitizer moiety converted to heat; heat disrupts thermo-labile bonds or induces conformational change in gatekeepers	Photothermal tumor ablation; spatiotemporally precise drug release; theranostics	[37,52]
Magnetic hyperthermia	Alternating magnetic field (AMF)	Superparamagnetic iron oxide NPs (SPIONs)	AMF induces Néel/Brownian relaxation in magnetic cores, generating local heat that cleaves thermolabile bonds or disrupts lipid bilayers	Deep-tissue tumor therapy; MRI-guided drug release; combination with chemotherapy	[36]
Ultrasound-mediated	Focused ultrasound + microbubble cavitation	Drug-loaded microbubbles; echogenic liposomes	Acoustic cavitation transiently increases membrane permeability (sonoporation), enabling enhanced NP cellular uptake and focal drug delivery	Blood-brain barrier opening; cardiac and tumor delivery; non-invasive, focal drug activation	[53]

**Table 3:** Representative clinically approved and late-stage nanoparticle drug delivery products: platform type, active ingredient, indication, approval status, and key clinical benefit.

Product/Platform	NP Type	Active Ingredient	Indication	Approval Status	Current Market/Clinical Status	Key Clinical Benefit	Ref.
Doxil^®^/Caelyx^®^	PEGylated liposome	Doxorubicin	Ovarian cancer, Kaposi’s sarcoma	FDA-approved (1995)	Currently marketed	Reduced cardiotoxicity vs. free doxorubicin; enhanced tumor accumulation via EPR effect	[13]
Abraxane^®^	Albumin-bound NP	Paclitaxel	Breast, lung, pancreatic cancer	FDA-approved (2005)	Currently marketed	Eliminates Cremophor^®^ vehicle toxicity; improved tolerability; higher dose delivery	[13]
Onpattro^®^ (patisiran)	Ionizable lipid NP	siRNA (transthyretin)	Hereditary transthyretin amyloidosis	FDA-approved (2018)	Currently marketed	First approved siRNA therapeutic; demonstrated liver-targeted gene silencing in humans	[8,31]
Comirnaty^®^/Spikevax^®^	Ionizable lipid NP (LNP)	mRNA (SARS-CoV-2 spike)	COVID-19 prevention	FDA-approved (2021)	Currently approved; updated formulations remain in clinical use	First approved mRNA vaccines; validated LNP as clinical nucleic acid delivery platform	[63,64]
DepoCyt^®^ (liposomal cytarabine)	Sustained-release liposome	Cytarabine	Lymphomatous meningitis	FDA-approved (1999)	Discontinued in the USA; marketing authorization withdrawn in the EU (2018)	Extended CSF drug residence time; reduced injection frequency from every 4 days to every 2 weeks	[43]
Vyxeos^®^ (CPX-351)	Liposome (fixed molar ratio)	Daunorubicin + cytarabine	Acute myeloid leukemia (AML)	FDA-approved (2017)	Currently marketed	Synergistic dual drug delivery at fixed ratio; improved OS vs. conventional 7 + 3 regimen in high-risk AML	[13]
Gold NP photothermal (AuroLase^®^)	Silica-gold nanoshells	Photothermal agent	Prostate cancer (ablation)	Investigational; evaluated in clinical studies	Investigational; not FDA-approved for prostate cancer treatment	Focal tumor ablation using NIR laser; minimally invasive; targeted tissue destruction with thermal mapping	[37]

**Table 4:** AI and machine learning approaches in nanoparticle drug delivery research: specific application, representative system, and key outcomes.

AI/ML Approach	Application in Nanomedicine	Representative System	Key Outcome	Ref.
Gradient boosting/LightGBM	Predict nanoparticle size, PDI, encapsulation efficiency from formulation inputs; screen polymer–lipid ratios	TuNa-AI	Reduced excipient usage by 75% in a trametinib formulation while maintaining in vitro efficacy and in vivo pharmacokinetics	[67]
Multi-view learning (neural networks)	Predict in vivo pharmacokinetics (AUC, Cmax, clearance) from physicochemical descriptors and animal model data	AI-PK model	Achieved high predictive accuracy for PK parameters from NP surface/size descriptors across multiple NP classes	[68]
Random forest/ensemble methods	High-throughput toxicity screening; predict cytotoxicity and genotoxicity from structural and surface descriptors	Nanotoxicology ML panel	Enables multi-variable safety assessment prior to synthesis, replacing one-at-a-time experimental testing	[71]
Convolutional neural networks (CNNs)	Image-based phenotypic analysis of NP-treated cells; pulmonary nodule malignancy classification from CT scans	AI-aided radiomics + adaptive NP surface design	Demonstrated automated lesion characterization enabling patient-specific NP surface modification strategy	[3,46]
In silico digital twins (ISTs)	Patient-specific simulation of NP pharmacokinetics, dose-response, and treatment optimization before clinical administration	IST platform	Personalizes NP dosing regimen based on patient age, genetics, organ function; reduces trial-and-error in clinical settings	[72]
Protein structure diffusion (RFdiffusion)	De novo design of targeting ligands and antibody fragments with optimized affinity for disease-associated receptors	RFdiffusion-guided ligand design	Enables rational design of targeting moieties with defined binding specificity; reduces reliance on empirical ligand screening	[70]
Microfluidics-coupled AI	Real-time process control of NP synthesis; adaptive feedback for size and PDI control during LNP manufacturing	AI-integrated microfluidic platform	Rapid formulation optimization; consistent particle quality; scalable integration with industrial LNP manufacturing	[41]

**Table 5:** Critical barriers to the clinical translation of nanoparticle drug delivery systems: root cause, current mitigation approaches, and development status.

Challenge	Root Cause	Current Mitigation Strategies	Development Status	Ref.
Batch-to-batch irreproducibility	Sensitivity of NP formation to mixing conditions, temperature, and reagent quality; lack of standardized protocols	Microfluidic manufacturing with real-time process control; AI-assisted parameter optimization; critical quality attribute (CQA) frameworks	Microfluidic scale-up demonstrated for LNPs; ISO and ICH Q8/Q10 frameworks being adopted; industrial IJM systems achieving consistent output	[28,41,42]
Protein corona formation	Serum proteins adsorb onto NP surface upon systemic injection, altering targeting ligand accessibility and accelerating clearance	PEG and zwitterionic stealth coatings; biomimetic cell-membrane cloaking; corona-resistant surface chemistries	PEGylation well-established but anti-PEG antibody formation reported; RBC membrane and CD47 ‘don’t-eat-me’ signals under active investigation	[7,14,15]
Inconsistent EPR-mediated targeting	EPR effect heterogeneous across tumor types, patient populations, and vascularization states; reduced in clinical vs. preclinical settings	Active targeting with receptor-specific ligands, antibodies, aptamers; multi-ligand systems targeting >1 receptor; tumor-penetrating peptides	Active targeting demonstrated in preclinical models; clinical benefit over passive targeting remains under investigation; EPR debate ongoing	[2,29]
Limited CNS penetration	Blood–brain barrier (BBB) restricts most NPs; tight junctions exclude particles >8–10 nm by paracellular transport	Receptor-mediated transcytosis (transferrin, LDL); focused ultrasound + microbubbles for transient BBB opening; cell-membrane-coated NPs	Focused ultrasound-mediated BBB opening evaluated in Phase I/II clinical trials, including recurrent glioblastoma (NCT03744026); no clinically approved CNS nanoparticle product yet	[32,55,76]
Poor nucleic acid stability	Serum nucleases rapidly degrade unprotected RNA/DNA; endosomal trapping limits intracellular delivery efficiency	Ionizable lipid NPs with pH-responsive endosomal escape; chemical modification (2′-OMe, phosphorothioate); polymeric encapsulation	Demonstrated in approved Onpattro^®^ and mRNA vaccines; liver targeting well-established; extrahepatic delivery still challenging	[8,30,31]
Manufacturing scale-up cost	High-resolution fabrication (plasma etching, CVD, lithography) incompatible with commercial volume production; high raw material costs	Roll-to-roll processing; impingement jet mixing (IJM); low-cost biopolymer substrates; green solvent synthesis	IJM demonstrated for LNP scale-up; biopolymer platforms in early development; full commercial scalability not yet universally demonstrated	[28,42]
Long-term safety and biodistribution	Unclear fate of non-degradable inorganic NPs (Au, Fe_3_O_4_) after repeated dosing; organ accumulation in liver/spleen; degradation product toxicity	Biodegradable coating strategies; comprehensive multi-organ toxicokinetic studies; AI-assisted toxicity prediction before synthesis	Extensive preclinical safety data required by FDA/EMA; standardized nanotoxicology protocols under development by ISO/OECD	[16,17]
Regulatory classification ambiguity	NPs classified differently (drug, biologic, device) depending on mode of action; nucleic acid NPs act as both API and excipient simultaneously	Harmonized nanotechnology-specific guidelines (FDA guidance 2022; EMA reflection paper); International harmonization via ICH	FDA and EMA guidance documents published; nucleic acid NP regulatory pathway being actively debated post-COVID vaccine approval	[18,74]

**Table 6:** Green and sustainable manufacturing strategies for nanoparticle drug-delivery systems: approach, sustainability advantage, therapeutic relevance, and key findings.

Approach	Sustainable Strategy	Drug-Delivery Relevance	Key Findings	Ref.
Green-solvent PLGA nanoparticle synthesis	Replacement of conventional hazardous/petroleum-derived solvents with safer, more sustainable solvents	PLGA nanoparticles for therapeutic delivery	Green-solvent formulations produced stable PLGA nanoparticles with physicochemical properties and cellular uptake comparable to conventionally prepared nanoparticles	[81]
Sustainable PLGA formulation	Use of green/alternative solvents in nanoprecipitation and nanoemulsion processes	Biodegradable PLGA drug-delivery systems	Alternative solvents can reduce reliance on hazardous organic solvents while maintaining nanoparticle formation and relevant particle characteristics	[82]
Microfluidic PLGA nanoparticle production	Reduced material, energy, and reagent consumption through controlled microscale processing	Controlled production of PLGA drug carriers	Microfluidic processing provides improved control over particle properties while reducing material, energy, and processing requirements	[83]

## Data Availability

No new data were created or analyzed in this study. Data sharing is not applicable to this article.
